# Current status and prospects of genetically modified porcine-to-human cardiac xenotransplantation

**DOI:** 10.1007/s10047-025-01504-z

**Published:** 2025-05-04

**Authors:** Takuji Kawamura, Shunsuke Saito, Takura Taguchi, Daisuke Yoshioka, Ai Kawamura, Yusuke Misumi, Takashi Yamauchi, Shuji Miyagawa, Shigeru Miyagawa

**Affiliations:** 1 Department of Cardiovascular Surgery, Osaka University Graduate School of Medicine, 2-2 Yamada-Oka, Suita, Osaka 565-0871 Japan; 2 Department of Pediatric Surgery, Osaka University Graduate School of Medicine, 2-2 Yamada-Oka, Suita, Osaka 565-0871 Japan

**Keywords:** Cardiac xenotransplantation, Genetic engineering, Pig, End-stage heart failure

## Abstract

Cardiac xenotransplantation utilizing genetically modified pigs presents a promising avenue for treating end-stage heart failure, a leading cause of mortality worldwide. This paper delineates the current landscape of heart failure treatment in Japan, emphasizing the limitations of existing therapies such as heart transplantation and implantable left ventricular assist devices. It discusses the history and advancements in the development of genetically modified pigs for xenotransplantation, highlighting recent breakthroughs and challenges. The manuscript also addresses the specific challenges facing the implementation of xenotransplantation in Japan, including the selection of suitable genetically modified pigs, ensuring organ safety, patient selection criteria, transplantation protocols, and immunosuppression strategies. Drawing from international experiences and ongoing research efforts, the paper emphasizes the potential of xenotransplantation while acknowledging the hurdles that must be overcome for widespread clinical adoption.

## Introduction

In January 2022, a cardiac xenotransplantation using a genetically modified pig was performed on a 57-year-old male patient with end-stage heart failure at the University of Maryland, USA [1, 2], followed by a second case in a 58-year-old male patient in September 2023 [3]. This groundbreaking event has introduced a promising new treatment option for heart failure, the leading cause of death worldwide. In Japan, heart failure is the second leading cause of death, and the number of patients is expected to increase due to the aging population [4]. Therefore, the development and introduction of cardiac xenotransplantation as a new treatment option is of crucial importance. This paper introduces the current status of heart failure treatment in Japan, the history and current status of the development of genetically modified pigs for xenotransplantation, and presents future challenges.

## Current status of treatment of severe heart failure

To make xenotransplantation using genetically modified pigs a widespread medical treatment, it is crucial to consider the types of patients who should be indicated for cardiac xenotransplantation. Two cardiac xenotransplantations performed after 2022 were approved by the Food and Drug Administration (FDA) for patients with end-stage heart failure who were ineligible for heart transplantation or implantable ventricular assist device placement, and for whom experimental xenotransplantation was anticipated to provide non-inferior outcomes compared to continued treatment. When considering the current landscape of heart failure treatment, the indication for xenotransplantation, including its potential application in pediatric patients, is generally considered for those unable to undergo conventional cardiac replacement therapies and whose life expectancy is estimated to be only a few months with the available treatment options. To further elucidate the current indications for xenotransplantation, this review first outlines the status of severe heart failure management in Japan and examines potential candidates for this innovative therapeutic approach.

The treatment of heart failure is considered according to the clinical stage of the disease. Patients whose heart failure symptoms are difficult to control after lifestyle management, drug treatment, and therapeutic intervention for valvular disease and coronary artery lesions are classified as Stage D, as defined by the American College of Cardiology, and are indicated for heart transplantation or an implantable left ventricular assist device (LVAD) to improve life expectancy. In Japan, the indication for heart transplantation is defined as preferably being under 65 years of age, and many patients are not indicated due to exclusion criteria such as dysfunction of organs other than the heart, active infection, or malignancy. Additionally, according to a report by the Japanese Heart Transplant Study Group, the average waiting period for a heart transplant is around five years, which is a very long waiting period compared to other countries. During this prolonged waiting period for heart transplantation, some patients do not survive due to various complications. Furthermore, implantable LVADs can either be used as a bridge to heart transplantation or as destination therapy, both of which require caregivers, and there are patients who are socially excluded from these treatments. Additionally, implantable ventricular assist devices are currently limited to use as left ventricular support, limiting their effectiveness in treating biventricular failure with right heart failure, constrictive cardiomyopathy, and hypertrophic cardiomyopathy, the primary pathology of which is diastolic failure. Even in patients who are able to use an implantable LVAD, complications such as cerebrovascular disease and infections require constant attention. In summary, heart transplantation and implantable LVADs are currently available as options for the treatment of severe stage D heart failure with a promising long-term prognosis, but it is certain that there are patients who cannot be treated due to the limited indications and complications of both options.

The prognosis of such patients with severe stage D heart failure who are not eligible for heart transplantation or implantable LVAD has been reported as follows [5]. In brief, the prognosis of 35 patients with severe heart failure who were treated with temporary mechanical support between 2010 and 2022 at our institution but who were not eligible for cardiac transplantation or implantable cardiac replacement was investigated, with a median survival of 3.2 months, a 6-month survival rate of 32%, and a 1-year survival rate of 15%. The indication for cardiac xenotransplantation in this poor prognosis patient group should be determined after careful consideration of whether cardiac xenotransplantation is a promising option for improving the prognosis of life.

## History of cardiac xenotransplantation development

The first reported xenotransplantation of a heart from a non-human animal was performed in 1964 by Hardy et al [6]. in a case of orthotopic transplantation from a chimpanzee, who died of rejection one hour after the operation. Since then, several cases of concordant transplantation from chimpanzees, baboons, and other primates, and discordant transplantation from pigs and sheep have been reported [7], but they were not successful enough to become widespread in clinical practice. With the development and progress of allogeneic heart transplantation and LVADs, attention and expectations for xenotransplantation relatively decreased.

However, the pig was judged to be the most suitable donor for xenotransplantation in terms of size, reproductive supply, risk of transmission to humans, and animal welfare, and the development of xenotransplantation has progressed with the development of genetic engineering. First, from the elucidation of the mechanism of rejection, discrepancies in the response of the complement system between pigs and humans were reported, and then species-specific carbohydrate antigens (αGal, Neu5Gc) or a blood group antigen (SDa) were reported that serve as antigens between pigs and humans [8, 9]. This led to attempts to create genetically modified pigs that are less susceptible to rejection using genetic engineering methods, i.e., transgenic (TG) and genetic knockout (KO).

By the early 2000s, genetically modified pigs with TGs for human complement regulators such as membrane cofactor protein (MCP; CD46), decay accelerating factor (DAF; CD55), and CD59 had been created [10–15] and reported reduced rejection of heterotopic and orthotopic heart transplants and extended graft engraftment [9].

Various methods have been tried for KO of carbohydrate antigens in pigs, as ES cells have not been established in pigs, and in 2002, a genetically modified pig with KO of αGal glycosyltransferase was generated [16, 17]. In addition to these, other gene-modified pigs have been created, such as CD47-TG for controlling rejection by the innate immune system via macrophages, coagulation regulator-TG (thrombomodulin, protein C receptor), and growth factor receptor-KO for suppressing transplant cardiac hypertrophy. Currently, gene-modified pigs combining KO and TG of these multiple genes are used for clinical applications.

With the creation of genetically modified pigs, the survival time of transplanted organs in preclinical xenotransplantation into primates has increased. Until the early 2000s, grafts were only engrafted for a few minutes to a few days [18], but the creation of pigs with multiple gene modifications and improved immunosuppressive drugs has enabled graft engraftment for up to 945 days for heterotopic heart transplants [19] and up to 264 days for orthotopic heart transplants [18]. The current consensus of the International Xenotransplantation Association is that for xenotransplantation to be performed in clinical practice, genetically modified pigs and transplant teams that can achieve a minimum of six months of orthotopic graft engraftment are required.

## Advancements in genetic modification of pigs for xenotransplantation

Significant progress has been made in the genetic modification of pigs for xenotransplantation, particularly through the knockout of xenogeneic antigens and the expression of immunoregulatory molecules. The genetically engineered pigs currently used in clinical practice feature the following modifications:

- Knockout (KO) of carbohydrate antigens (αGal, Neu5Gc, SDa) to reduce hyperacute rejection [20].
- Introduction of human complement regulatory proteins (CD46, CD55, CD59) to mitigate immune response [21].
- Expression of human thrombomodulin and endothelial protein C receptor to improve coagulation compatibility [22, 23].
- CD47 overexpression to prevent macrophage-mediated rejection [24, 25].
- Targeted genetic modifications to address transplant-associated cardiac hypertrophy and regulate growth [26].

Pigs used in recent clinical xenotransplantation trials, such as those by Revivicor in the United States, incorporate ten or more genetic modifications. Understanding these advancements is crucial for Japan’s consideration of whether to introduce existing genetically modified pigs or develop an original domestic model.

## The role of perfusion preservation in xenotransplantation

**Fig. 1:**
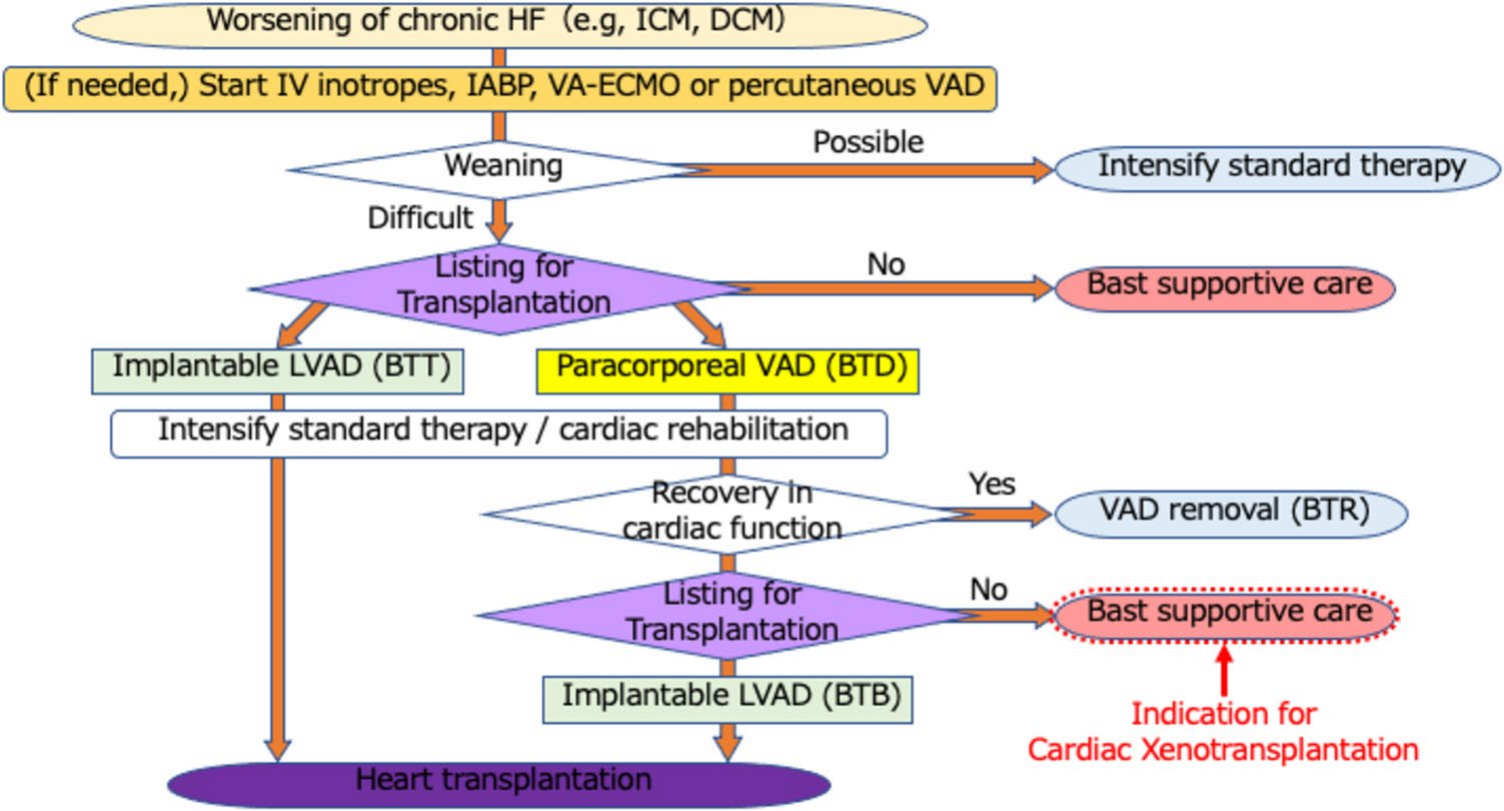
Flowchart of current acute heart failure treatment. Flowchart of severe heart failure treatment in Japan (prepared by the author based on the Guidelines for the Treatment of Acute and Chronic Heart Failure (2017 revision) (joint guidelines of the Japanese Society of Cardiology/Japan Heart Failure Society)) and patients indicated for allogeneic heart transplantation

Perfusion preservation has emerged as a critical factor in maintaining the viability of porcine hearts before transplantation [27]. Studies have demonstrated its efficacy in preserving graft function following cardiac xenotransplantation [28] and it has been successfully employed in clinical applications [1]. Given the susceptibility of xenografts to ischemia–reperfusion injury, the integration of perfusion preservation techniques into xenotransplantation protocols is essential for optimizing outcomes (Fig. 1).

## Challenges for performing xenotransplantation in Japan

Since the implementation of xenotransplantation using gene-modified pigs at the University of Maryland, USA, in January 2022, a consortium of experts from various fields has been formed to study the feasibility of xenotransplantation in Japan. The following is a list of issues that have been raised as problems that need to be solved.

(i) Selection of types of genetically modified pigs.
  Although it should be considered whether to introduce a genetically modified pig that has been clinically applied in the USA or to develop it in Japan, it is likely to be quicker in terms of time to introduce a genetically modified pig for which pre-clinical results have been obtained abroad. In that case, however, it is necessary to consider in what form (cells, fertilized eggs, frozen embryos, living organisms, etc.) it would be practical to introduce the gene-modified pigs from abroad. At the same time, it is also considered important to develop Japan’s original genetically modified pig that advances over the gene-modified pigs developed in other countries.
(ii) Ensuring the safety of organs removed from genetically modified pigs.
  It is a prerequisite that organs are extracted from Designated Pathogen-Free (DPF) pigs, although a definition of DPF in Japan needs to be formulated. As for zoonosis, it is necessary to consider pathogens that vary depending on the environment in which they grow, so it is considered necessary to formulate items unique to Japan with reference to examples from other countries. Additionally, it has been pointed out that human complement regulatory factors introduced to avoid immune reactions to xenotransplantation may act as receptors for viruses that do not originally infect pigs [9, 29–31]. It is also considered necessary to pay attention to infectious diseases specific to genetically modified pigs for xenotransplantation purposes.
(iii) Selection of patients for xenotransplantation and establishment of treatment objectives.
  As mentioned above, the first indication for xenotransplantation is considered to be patients who are not eligible for existing allogeneic heart transplantation or an implantable ventricular assist device and who are currently under the Best Supportive Care (BSC) strategy. Naturally, patients in poor general condition, which affects the prognosis after transplantation, should be avoided, and patients whose condition is stable to some extent but who cannot be discharged from hospital with extracorporeal assisted circulation should be treated with the aim of discharge or used to improve quality of life, even if they cannot be discharged. It may also be used as a bridge to allogeneic heart transplantation for patients who are eligible for allogeneic heart transplantation but who cannot use an implantable ventricular assist device.
(iv) Protocols for organ removal, transport, and transplantation when performing allogeneic heart transplantation.
  Although related to (ii) above, it is considered that the location for the creation of genetically modified pigs and organ procurement should be as close as possible to the facility where the transplantation operation is performed. In practice, it is assumed that transplant facility staff will go to the place where organs are extracted and transport the organs after the procurement, as is currently the case with allogeneic heart transplants. Additionally, it is necessary to consider the anatomical differences between porcine and human hearts present unique challenges in xenotransplantation. Porcine aortic, pulmonary artery, and superior and inferior vena cava diameters are generally smaller, with shorter branch distances in the aorta and superior vena cava, while the inferior vena cava is comparatively longer. Notably, the angle between the ascending and descending aorta is approximately 90 degrees in pigs, compared to 180 degrees in humans [32, 33]. These anatomical disparities necessitate careful selection of anastomotic techniques during transplantation. The biatrial anastomosis method has been suggested as a preferable alternative to bicaval anastomosis, potentially mitigating the risk of superior and inferior vena cava kinking [34]. Furthermore, to address size discrepancies in aortic and pulmonary artery anastomoses, excising the donor pig heart’s vessels at a more peripheral site, thereby increasing the vessel diameter, has been proposed. Regarding left atrial size mismatch, a technique involving the excision of the donor pig heart’s left atrium up to the region between the left and right pulmonary veins has been reported, allowing for a more favorable anastomosis [34]. These adaptations are critical for ensuring optimal graft function and long-term outcomes in cardiac xenotransplantation.
(v) Formulation of immunosuppression protocols.
  The optimization of immunosuppressive therapy has been a key factor contributing to improved outcomes in xenotransplantation. A combination of calcineurin inhibitors (e.g., tacrolimus, cyclosporine), costimulatory blockade agents (anti-CD40 or anti-CD154 antibodies), mycophenolate mofetil, corticosteroids, and targeted coagulation management has demonstrated significant prolongation of graft survival. In recent clinical xenotransplantation cases, such as those conducted at the University of Maryland, the immunosuppressive regimen included anti-CD40 monoclonal antibodies, representing a major breakthrough in preventing rejection [1]. While the use of anti-CD154 or alternative anti-CD40 antibodies has shown promise, these agents are not yet approved for clinical application and would require regulatory authorization for use in xenotransplantation. Further refinement of immunosuppressive protocols, tailored to the specific clinical and regulatory environment in Japan, is essential to ensure optimal graft outcomes and patient safety.

## Concluding remarks

With the increasing number of heart failure patients, there are high expectations for cardiac xenotransplantation. In May 2023, our department had the very valuable opportunity to hear Dr. Muhammad Mohiuddin of the University of Maryland, who led the aforementioned xenotransplantation using genetically modified pigs in 2022, give a lecture at Osaka University on the clinical situation of xenotransplantation. He emphasized that patients who had undergone cardiac xenotransplantation regained consciousness and were able to meet their families and that the medical team was thanked by everyone involved. Although the results of the treatment are not yet convincing and there are still high barriers to overcome before it can become widespread clinical practice, it is clear that there are patients who can be saved, depending on where the objectives are set. The motivation for the pioneering work of a doctor at the forefront of the field is simple, and we were impressed by his strong passion and the need to tackle the issues raised in this presentation with sincerity. Additionally, while the environment surrounding xenotransplantation is changing with the times, it is impossible to have high expectations for clinical application without the basic researchers who have continued basic research even when it was not the focus of attention and who have steadily studied the molecular mechanisms of rejection. We would like to pay tribute to their passion and efforts.
